# Chagas disease reactivation associated with cutaneous vasculitis in a heart transplant patient

**DOI:** 10.1016/j.jdcr.2023.09.031

**Published:** 2023-10-11

**Authors:** Mark Biro, Alison L. Hill, Michael Cardis, Helena B. Pasieka, Freba Z. Farhat

**Affiliations:** a Department of Dermatology, MedStar Washington Hospital Center, Washington, District of Columbia; b Department of Dermatology, Georgetown University School of Medicine, Washington, District of Columbia; c Departments of Dermatology & Medicine, Uniformed Services University, Bethesda, Maryland

**Keywords:** chagas, chagas disease, chagas disease secondary to immunocompromised status, heart transplant, panniculitis, transplant

## Introduction

Chagas disease is a parasitic infection caused by *Trypanosoma cruzi* (*T cruzi*) that is transmitted by several species of triatomine bugs native to Latin America.^1^, ^2^, ^3^ While most acute infections are asymptomatic, a subset of patients develop cutaneous manifestations including Romaña’s sign or a chagoma.^1^, ^2^, ^3^ Following the acute phase most patients remain chronically infected but are asymptomatic. A subset of these patients develop severe complications, including Chagas cardiomyopathy which may require cardiac transplantation.^3^^,^^4^ Following cardiac transplantation immunosuppression places these individuals at risk for *T cruzi* reactivation.^1^, ^2^, ^3^, ^4^, ^5^, ^6^, ^7^ Subcutaneous nodules and plaques, often associated with underlying panniculitis, represent the primary cutaneous manifestation of Chagas disease reactivation in transplant patients.^1^ As Chagas disease reactivation may be associated with additional life threatening complications in immunosuppressed patients, recognition of disease reactivation is critical. We present a case of disseminated panniculitis associated with cutaneous vasculitis in a heart transplant patient representing the earliest manifestation of Chagas disease reactivation.

## Case report

**Fig 1:**
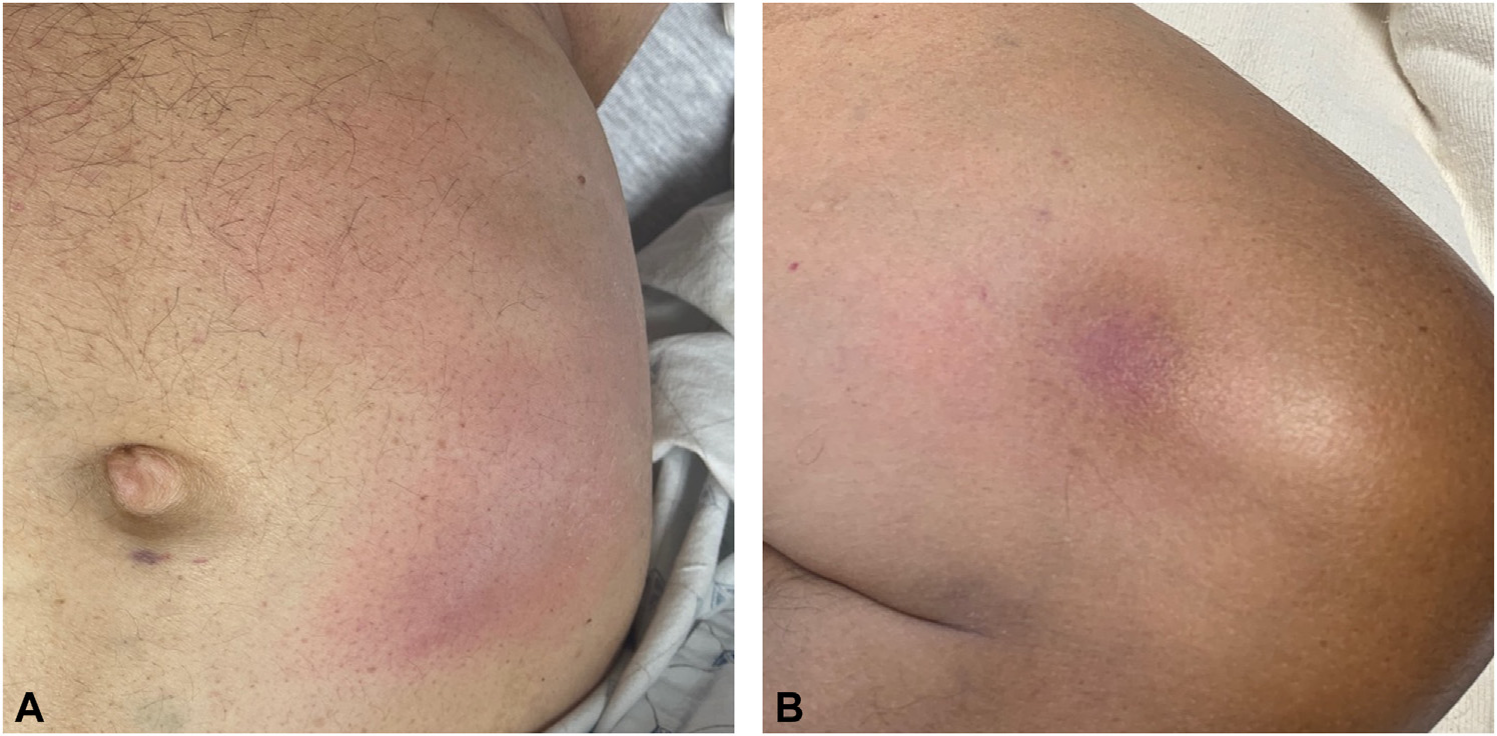
**A** & **B,** Clinical presentation demonstrating multiple, ill-defined, erythematous to dusky, indurated, tender, plaques.

**Fig 2:**
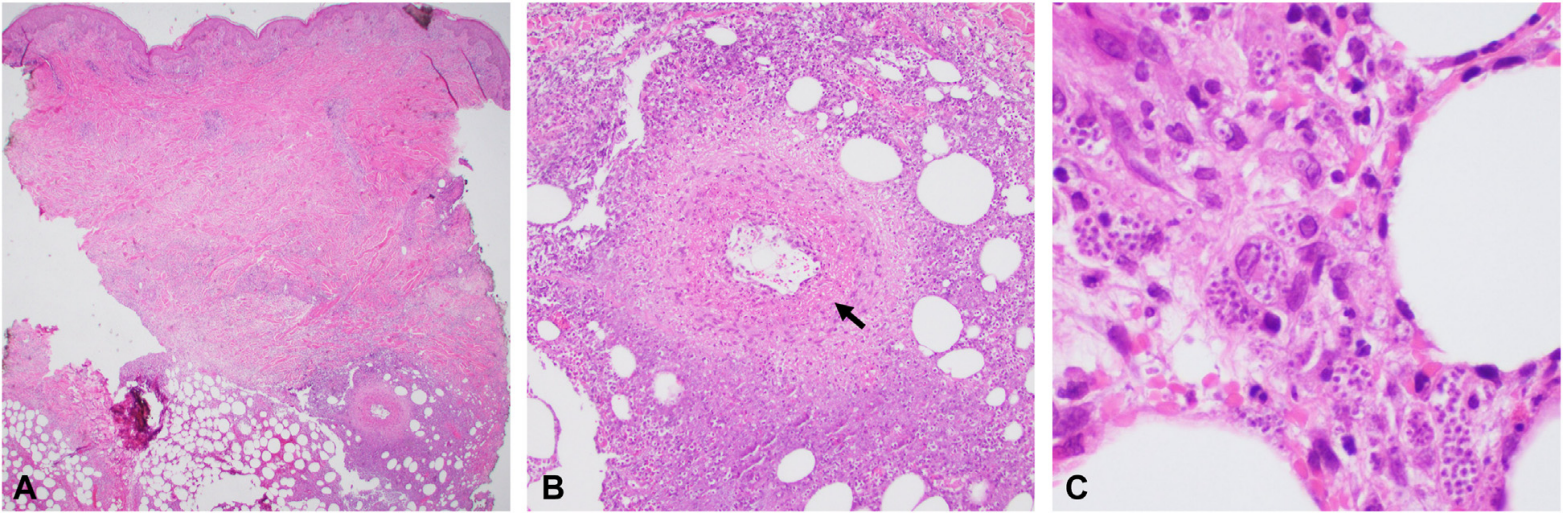
**A,** Low power photomicrograph demonstrating deep dermal to subcutaneous infiltrate. **B,** Medium-vessel vasculitis, denoted by *arrow*, with lobular panniculitis. **C,** Intracellular protozoal amastigotes, consistent with trypanosomiasis (**A, B,** and **C,** Hematoxylin-eosin stain; original magnifications: **A,** ×20; **B,** ×400; **C,** ×400).

A 61-year-old man developed indurated plaques involving the trunk and extremities, approximately 2 months after heart transplantation for Chagas cardiomyopathy. His posttransplant immunosuppression regimen included prednisone, tacrolimus, and mycophenolate mofetil. Exam demonstrated numerous poorly defined, warm, pink-to-violaceous, indurated, tender, nodules and plaques involving the abdomen, flanks, right thigh, and left knee (Fig 1, *A* and B). The patient had additional pertinent findings including fevers, tachycardia, and hypotension. Histopathological exam of a punch biopsy specimen from a lesion on the left thigh demonstrated deep dermal and subcutaneous inflammation with necrosis, medium-vessel vasculitis (Fig 2, *B*), and numerous intracellular protozoal amastigotes (Fig 2, *C*), supporting the diagnosis of Chagas disease reactivation. Subsequent serum PCR testing for *T cruzi* was positive. The skin lesions demonstrated marked improvement soon after starting a 3-month course of benznidazole.

## Discussion

Chagas Disease affects an estimated 7 million people worldwide,^8^ including 300,000 people in the United States.^5^ *T cruzi* is most often transmitted following a triatomine (also known as the “kissing bug”) bite. When the infected insect bites the host, trypomastigotes are excreted in feces and enter the uninfected host at the bite wound or nearby mucous membrane. Trypomastigotes subsequently invade host cells where they differentiate into amastigotes and undergo replication. Upon completion, amastigotes differentiate into trypomastigotes, cause cell lysis, and then spread through the bloodstream and lymphatics, preferentially invading the heart and gastrointestinal tract.^2^ Additional vectors of transmission include blood transfusion, vertical transmission in pregnancy, as well as bone marrow and solid organ transplantation.^2^

Chagas disease consists of acute and chronic phases. Following an incubation period of 1 to 2 weeks, the acute phase is characterized by detectable parasitemia. Most patients are asymptomatic; however, a subset of patients develop cutaneous manifestations. When patients develop a red, indurated skin nodule at the site of inoculation, it is called a chagoma. Biopsy of a chagoma demonstrates intracellular protozoal amastigotes, similar Chagas disease reactivation. When patients develop eyelid edema and associated with conjunctivitis near the site of inoculation, it is called Romaña’s sign.^1^^,^^2^ Finally, a small minority of patients will develop a diffuse morbilliform eruption during an acute infection, known as schizotrypanides.^1^ In immunocompetent individuals, parasitemia resolves within 2 months and thereafter most patients remain chronically infected but asymptomatic.^2^^,^^3^

Approximately 20% to 30% of patients develop Chagas cardiomyopathy, 10 to 40 years after initial infection, which may require cardiac transplantation.^2^, ^3^, ^4^ Chagas disease may reactivate in severely immunosuppressed patients, including solid organ transplant patients or patients with human immunodeficiency virus.^1^^,^^2^ The most common systemic manifestations of Chagas disease reactivation include fever, myalgias, meningoencephalitis, myocarditis, cardiac arrythmias, and cardiogenic shock.^1^, ^2^, ^3^, ^4^^,^^6^^,^^7^ Our patients’ symptoms of fever, tachycardia, and hypotension were initially suggestive of an alternate diagnosis, like bacterial infection. However, unlike bacterial infection, Chagas disease reactivation will not respond to antibiotic agents. Upon review of the literature, the primary cutaneous manifestation of Chagas disease reactivation includes development of tender, subcutaneous nodules and plaques, commonly with underlying panniculitis, involving the trunk and extremities, which may ulcerate.^1^^,^^2^^,^^9^^,^^10^ A review of histopathologic findings of 7 transplant patients with Chagas disease reactivation demonstrated the most common biopsy findings included presence dermal and interstitial amastigotes.^9^ Our case represents a unique presentation of Chagas disease reactivation associated with panniculitis and underlying cutaneous vasculitis, which has not been previously reported.

Once Chagas reactivation is confirmed patients are treated with antitrypanosomal therapy with either benznidazole or nifurtimox.^2^^,^^6^^,^^7^ Benznidazole is considered first-line therapy in patients with Chagas disease given its more favorable side effect profile compared to nifurtimox. The total length of therapy for either agent ranges from 60 to 90 days.^1^^,^^2^^,^^7^ Our patient's response to therapy suggests that benznidazole therapy may alone be adequate to treat Chagas disease reactivation and associated vasculitis, even in the setting of immunosuppression.

In conclusion, chronic Chagas disease reactivation may occur in solid organ transplant patients. Although there are no formal guidelines for the monitoring of Chagas disease in organ transplant patients, awareness is critical, as there may be concurrent life-threatening complications, including arrhythmias, myocarditis, meningitis, and encephalitis.^1^^,^^2^^,^^6^^,^^7^ In such patients, cutaneous involvement may present as subcutaneous nodules or plaques with underlying panniculitis. Our case demonstrated additional underlying cutaneous vasculitis, which has not been previously reported. Diagnosis of Chagas disease reactivation may be confirmed by amastigotes on skin biopsy or polymerase chain reaction testing and allow for prompt initiation of antitrypanosomal therapy. Given the high risk for systemic complications, a multidisciplinary approach is recommended in the management of Chagas disease reactivation.

## Conflicts of interest

None disclosed.
